# Abnormal villous morphology mimicking a hydatidiform mole associated with paternal trisomy of chromosomes 3,7,8 and unipaternal disomy of chromosome 11

**DOI:** 10.1186/s13000-016-0471-9

**Published:** 2016-02-04

**Authors:** Neil J Sebire, Philippa C May, Baljeet Kaur, Michael J Seckl, Rosemary A Fisher

**Affiliations:** Trophoblastic Tumour Screening & Treatment Centre, Imperial College London, Charing Cross Campus, Fulham Palace Road, London, W6 8RF UK; Molecular Pathology Laboratory, Imperial College London, Hammersmith Campus, DuCane Road, London, W12 0NN UK

**Keywords:** Hydatidiform mole, dysmorphic villi, trisomy, paternal uniparental disomy, chromosome 11

## Abstract

**Background:**

Pregnancies affected by non-molar chromosomal abnormality may sometimes demonstrate abnormal chorionic villous morphology that is similar to partial hydatidiform mole. Determination of the underlying aetiology may be difficult in such cases.

**Case Presentation:**

This report describes a case referred to the regional trophoblastic disease unit as a possible hydatidiform mole that demonstrated both villous dysmorphology and abnormal p57^KIP2^ expression. Molecular genotyping revealed that while most chromosomes in the villous tissue were diploid and biparental, chromosomes 3, 7 and 8 were trisomic with an additional paternally derived chromosome. In contrast chromosome 11 showed uniparental disomy of paternal origin a situation more usually associated with complete hydatidiform moles. This unusual case highlights that exceptions may occur to the general rules of both histological morphology and immunoprofile, and that these can be resolved by detailed molecular genetic investigations.

**Conclusion:**

The findings confirm that trisomic pregnancies may demonstrate morphological villous features similar to hydatidiform mole, and that loss of p57^KIP2^ expression occurs due to an absence of maternally transcribed genes on chromosome 11 and can therefore be independent of androgenetic complete hydatidiform mole.

## Background

Hydatidiform moles (HM) are chromosomally abnormal pregnancies, characterised by relative overexpression of paternally derived genes, with abnormal chorionic villi and villous trophoblastic hyperplasia. Most HM are classified as complete HM (CHM) or partial HM (PHM), CHM being androgenetic diploid conceptions and PHM diandric triploids [1]. The diagnosis of CHM and PHM is usually based on histopathological examination of products of conception but, in around 10% of cases, such morphological findings may be non-diagnostic and ancillary investigations are required, including immunostaining and molecular genotyping [2–4]. The combination of villous dysmorphology with absent chorionic villous expression of p57^KIP2^ usually represents early CHM [5]. This report describes an unusual case with villous dysmorphology and negative p57^KIP2^ staining of villous cytotrophoblast and stroma in which the underlying diagnosis was triple trisomy with paternal uniparental disomy for chromosome 11. This case highlights both the clinical utility of molecular genotyping in this setting and furthermore provides confirmation of a mechanism for loss of p57^KIP2^ expression in non-molar gestations.

## Case presentation

### Clinical details

The patient was a 36 year-old female, G4P1, having had a normal live birth and two miscarriages. An ultrasound scan at 12 weeks gestation showed no embryo and raised the possibility of hydatidiform mole. Following evacuation of retained products of conception for a missed miscarriage, the patient was referred to the Trophoblastic Tumour and Screening Centre at Charing Cross Hospital with a provisional diagnosis of molar pregnancy. The patient’s serum hCG had reached normal by the time of registration and she remains well with no evidence of persistent trophoblastic neoplasia, delivering a healthy baby boy 29 months after referral to the centre.

### Histopathological examination

**Fig. 1:**
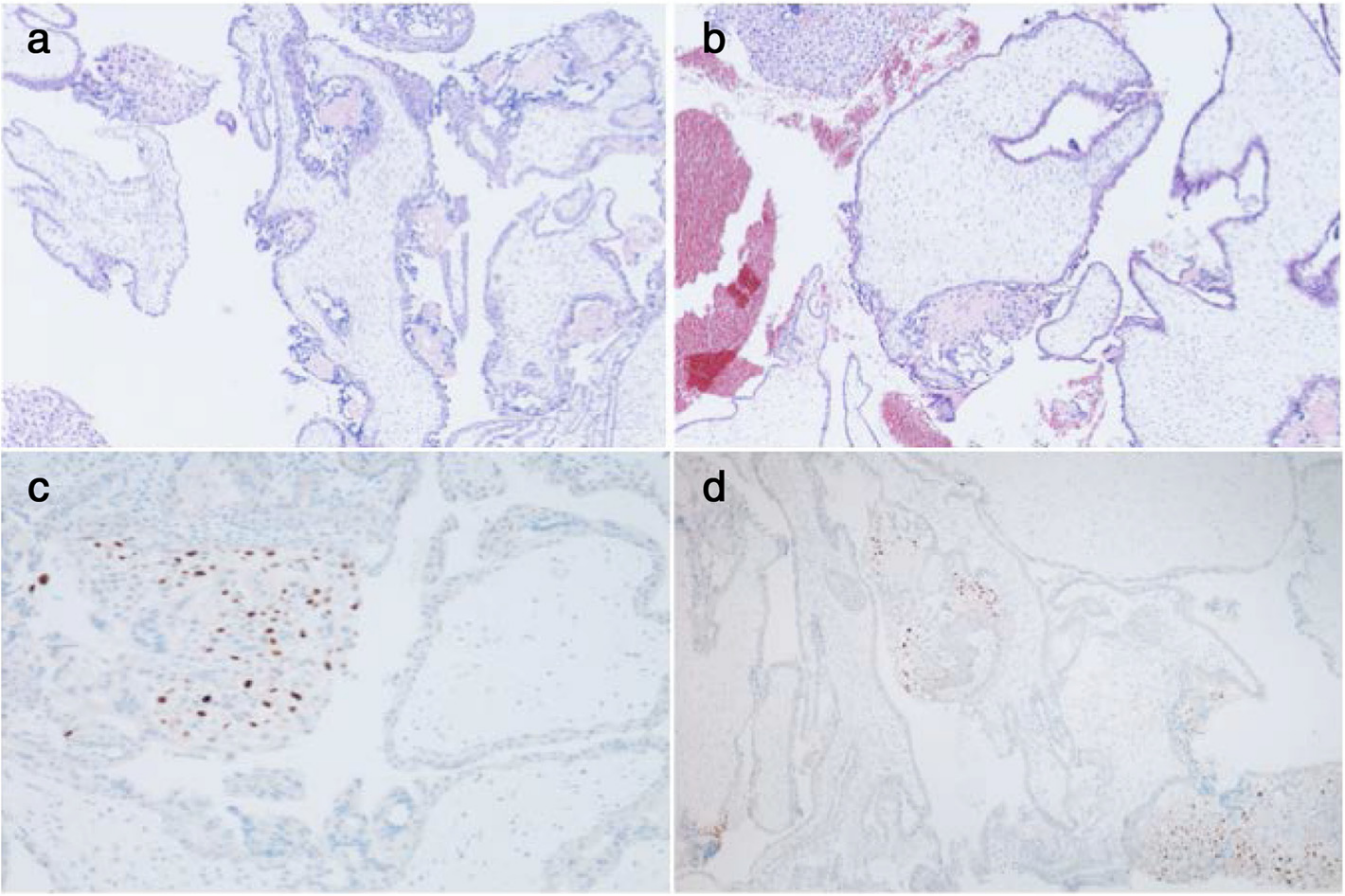
Photomicrographs of villous tissue showing haematoxylin and eosin staining and p57^KIP2^ immunostaining. **a** and **b**; (haematoxylin and eosin original magnification x40) demonstrate villous dysmorphism within the spectrum of that seen in molar pregnancies but not typical of complete or partial hydatidiform mole. **c** and **d**; (original magnification x100, immunostained with avidin-biotin detection system and haemtoxylin counterstain) show associated absence of villous p57^KIP2^ expression

Routine haematoxylin and eosin (H&E) stained 4 μm sections were reviewed, using published criteria for distinguishing PHM and CHM from non-molar miscarriage [6–8], by a specialist trophoblastic disease pathologist with more than 15 years experience in this field. Unstained 4 μm sections were immunostained using a mouse monoclonal primary antibody against p57^KIP2^ (Leica Biosystems, Newcastle) at a dilution of 1 in 50, and visualised using the Bond Polymer Refine Detection kit (Leica Biosystems, Newcastle) according to a standard protocol in a CPA approved diagnostic laboratory. Histological review demonstrated villous development consistent with early second trimester but with marked villous dysmorphic features, including variation in size and shape, irregular villous outlines with pseudoinclusions and patchy villous hydropic change closely mimicking hydatidiform mole but morphologically not diagnostic of either PHM or CHM. These changes were variable from field to field, with some more normal appearing villi and other areas with more prominent dysmorphism (Fig. 1). There was limited circumferential trophoblast hyperplasia relative to the degree of villous dysmorphism and no definite karyorrhexis was identified. The morphological differential diagnosis was between a HM and villous dysmorphism secondary to chromosomal abnormality. In view of the unusual morphology in this case, ancillary studies were undertaken including p57^KIP2^ immunostaining and molecular genotyping. P57^KIP2^ immunostaining showed normal strong nuclear expression in extravillous trophoblast fragments and decidua but with negative staining of villous cytotrophoblast and stroma (Fig. 1).

### Molecular genotyping

Initially five areas of placental tissue and a single area of maternal decidua were microdissected from formalin-fixed, paraffin-embedded (FFPE) pathological sections, with reference to a stained H&E section. DNA was then prepared from each sample using a QIAmp DNA FFPE Tissue Kit (Qiagen, Sussex, UK). DNA was subsequently prepared from 500 μl of paternal saliva using an Oragene DNA collection kit (DNA Genotek, Ontario, Canada). 1 μl DNA from the patient, her partner and villous tissue was amplified with primers for 15 short tandem repeat (STR) loci on 13 chromosomes, together with the amelogenin locus, using an AmpFlSTR Identifiler Plus kit (Applied Biosystems, Warrington, UK). PCR products were resolved by capillary electrophoresis using an ABI 3100 Genetic Analyser and genotypes determined using GeneMapper version 4.0 software (Applied Biosystems, Warrington, UK). In order to confirm the results obtained using the AmpFlSTR kit, and to investigate the origin of other chromosomes not represented in the kit, a panel of markers including STRs located on each chromosome was used to amplify DNA from the parental and placental samples, followed by analysis as described above.

**Fig. 2:**
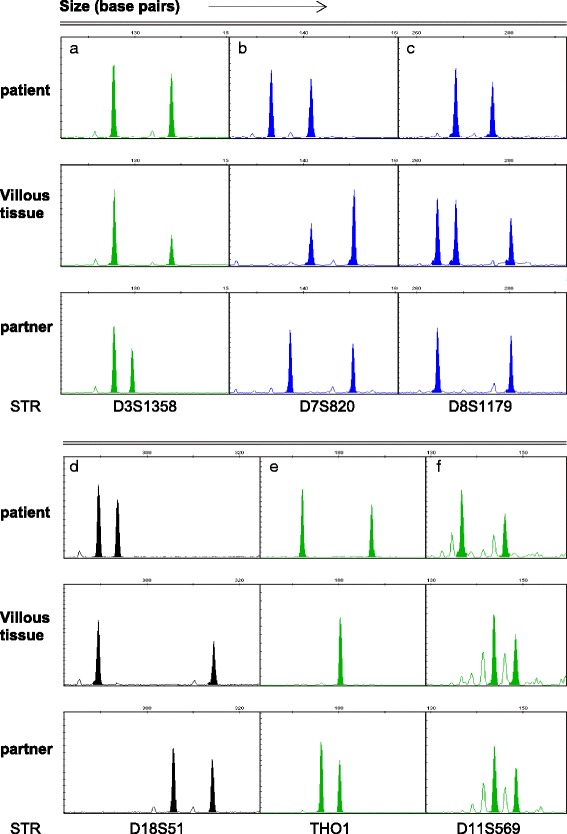
Partial genotypes of the patient, her partner and the villous tissue. **a**, **b** and **c** show genotypes in the parents and villous tissue following amplification of STR loci D3S1358, D7S820 and D8S1179 respectively. Trisomy of the villous tissue at these loci is demonstrated by the presence of two paternal alleles (**c**) or an increased dosage of a single paternal allele relative to the maternally derived allele (**a**, **b**). **d** shows an example of a disomic locus with one maternal and one paternal allele, found for loci on the majority of chromosomes. **e** and **f** represent loci on chromosome 11 for which the villous tissue has only paternally derived alleles

Fluorescent microsatellite genotyping of DNA from the parents and villous tissue, using the AmpFlSTR kit, revealed an unusual, but consistent, genotype in all DNA samples from the villous tissue (Table 1). Loci on chromosomes 3, 7, and 8 were trisomic, having three different alleles (D8S1179) or two alleles with one present at twice the dosage of the other (D3S1358; D7S820) (Fig. 2). Loci on chromosome 7 and 8 both had a single maternally derived allele and two alleles from the father while alleles at the D3S1358 locus were not informative with respect to origin. Six informative loci on chromosomes 4, 5, 12, 16 and 18 (Table 1; Fig. 2) exhibited equal parental contributions. Allele sizes for loci on 4 of the remaining chromosomes were consistent with an equal contribution from each parent (Table 1). However, the THO1 locus on chromosome 11 was androgenetic, having a single paternally derived allele (Fig. 2). There was no evidence of mosaicism in the tissue, the genotype of the villi being identical in all five areas of tissue examined. Further amplification with primers for STR loci on chromosomes 3, 7 and 8 confirmed trisomy for both the long and short arms of each chromosome and that the additional chromosome in each case was paternal in origin (Table 2). Amplification with primers for STR loci on the remaining autosomes and the X chromosome were consistent with an equal maternal and paternal contribution to the genome with the exception of chromosome 11. An absence of maternal alleles for six informative loci (Table 2) confirmed that the tissue was androgenetic for chromosome 11. While 4 loci were homozygous for a single paternal allele, the D11S569 and D11S934 loci were heterozygous demonstrating the involvement of two paternal copies of chromosome 11. The villous tissue therefore exhibited paternal disomy for chromosome 3, 7, 8 and 11 together with loss of maternal chromosome 11.

**Table 1:** Genotypes of parental and villous tissue for loci amplified by the AmpFlSTR kit

Locus	chr	Patient	Villi	Partner	Ploidy	Parental contributions
D8S1179	8	10 - 12	12 < 14	11 - 14	3n	M,P2,P2
D21S11	21	28 - 30	28	28 - 30	NI	NI
D7S820	7	9 - 11	8 - 9 - 12	8 - 12	3n	M,P1,P2
CSF1PO	5	10 - 11	10 - 12	11 - 12	2n	M,P
D3S1358	3	15 - 18	15 > 18	15 - 16	3n	NI
THO1	11	6 - 9.3	8	7 - 8	NI	P
D13S317	13	11 - 12	11 - 12	11	2n	NI
D16S539	16	13	11 - 13	11	2n	M,P
D2S1338	2	19 - 23	19 - 23	23 - 24	2n	NI
D19S433	19	14 - 15	14	14 - 15	NI	NI
vWA	12	17 - 18	16 - 18	16	2n	M,P
TPOX	2	8 - 11	8 - 11	8	2n	NI
D18S51	18	13 - 14	13 - 19	13 - 17	2n	M,P
AMEL		X	X	XY	NI	NI
D5S818	5	12 - 14	11 - 14	11	2n	M,P
FGA	4	21 - 28	21 - 22	22 - 24	2n	M,P

Alleles are numbered by comparison with the allelic ladder provided. chr, chromosome; M, maternal allele; P, paternal allele; P1, and P2, smaller and larger paternal allele respectively; NI, not informative

**Table 2:** Genotypes of parental and villous tissue for additional STR loci

Locus	chr	Patient	Villi	Partner	Ploidy	Parental contributions
Trisomic chromosomes						
D3S1300	3p	165 - 169	165 < 181	175 - 181	3n	M,P2,P2
D3S1314	3q	68 - 80	68 < 78	78 - 88	3n	M,P1,P1
D7S513	7p	152	136 - 140 - 152	136 - 140	3n	M,P1,P2
D7S669	7q	126	118 - 124 - 126	118 - 124	3n	M,P1,P2
D8S1786	8p	106	106 - 110 -116	110 - 116	3n	M,P1,P2
D8S1110	8q	270 - 274	258 - 270 - 274	258 - 270	3n	M,P1,P2
Disomic chromosome pairs with a maternal and paternal contribution						
D1S1656	1	136 - 161	141 - 161	141 - 150	2n	M,P
D2S1391	2	118 - 122	106 - 122	106 - 126	2n	M,P
F13A1	6	283 - 287	279 - 283	279 - 287	2n	M,P
D9S171	9	165 - 173	163 - 173	157 - 163	2n	M,P
D10S189	10	180 - 186	178 - 180	178 - 184	2n	M,P
D13S158	13	122	116 - 122	116	NI	M,P
D14S51	14	93 - 95	95 - 99	93 - 99	2n	M,P
D15S125	15	123 - 129	129 - 131	131 - 133	2n	M,P
D17S799	17	184	184 - 190	190	NI	M,P
D19S221	19	198 - 206	194 - 206	192 - 194	2n	M,P
D20S481	20	231	219 - 231	219 - 235	2n	M,P
D21S167	21	160 - 177	160 - 179	175 - 179	2n	M,P
D22S264	22	186 - 196	196 - 204	194 - 204	2n	M,P
DXS451	X	188	174 - 188	174	NI	M,P
Chromosome 11						
D11S922	11p	234 - 244	215	215 - 234	NI	P1
D11S2071	11p	170 - 189	194	166 - 194	NI	P2
D11S569	11p	137 - 146	144 - 148	144 - 148	2n	P1,P2
D11S916	11q	170 - 184	172	172	NI	P1
D11S925	11q	289	268	268	NI	P1
D11S934	11q	274 - 276	278 - 286	278 - 286	2n	P1,P2

Allele sizes are given in base pairs. chr, chromosome; M, maternal allele; P, paternal allele; P1, and P2, smaller and larger paternal allele respectively; NI, not informative

### Fluorescence in situ hybridisation

**Fig. 3:**
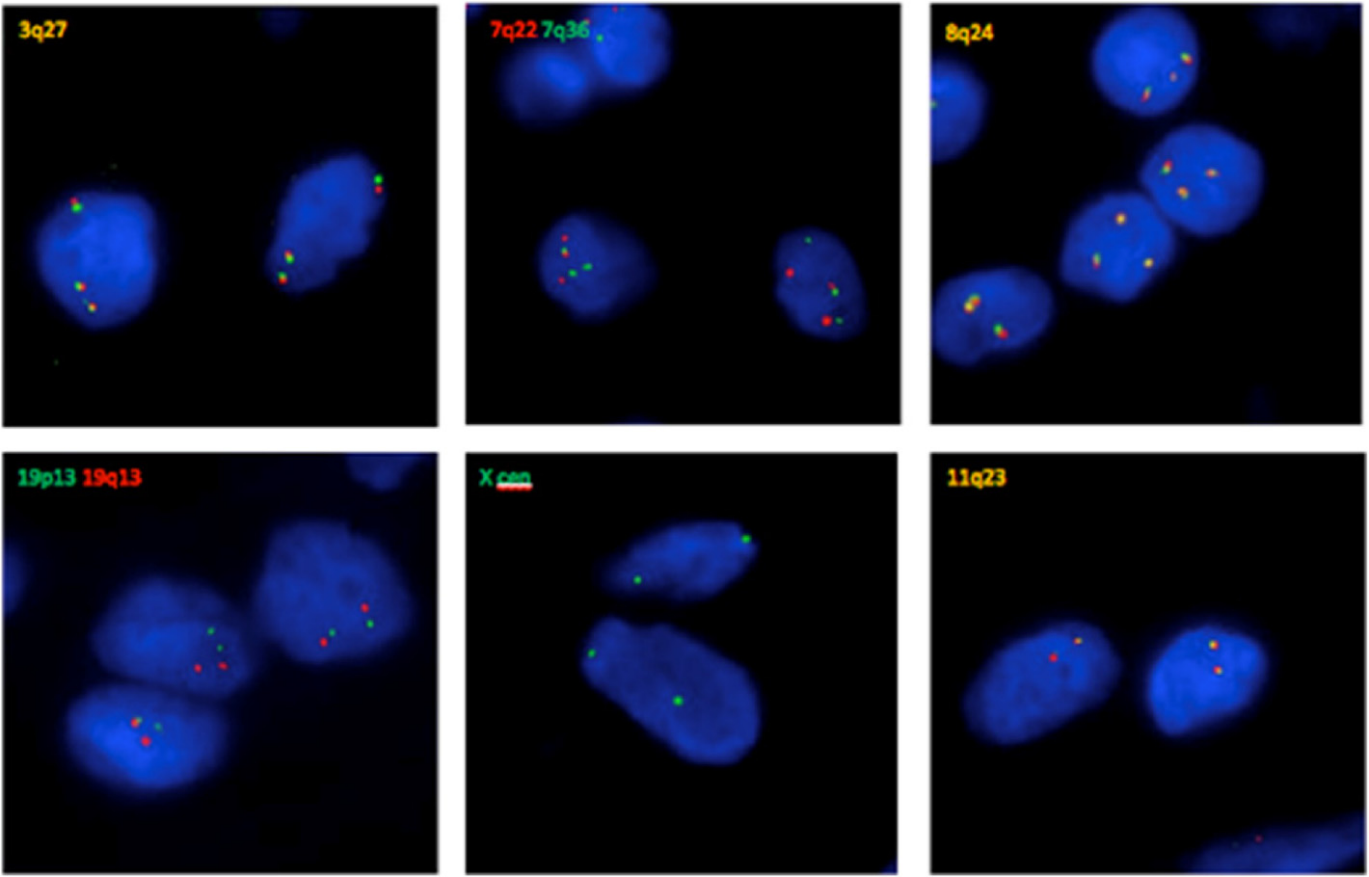
Interphase FISH analysis of representative villous cells. Probes for chromosomes 3, 7, and 8 (top panel) show one additional signal, consistent with trisomy for these chromosomes. Probes for chromosomes 11, 19 and X (lower panel) showed two signals only, consistent with disomy 11 and 19, and a female sex chromosome complement

In order to confirm the observations resulting from genotyping, fluorescence in situ hybridization (FISH) was performed on sections of the villous tissue. FFPE sections of the placental tissue were pretreated and hybridized using the ThermoBrite Elite according to manufacturer’s protocols (Leica Biosystems, Nussloch, Germany). FISH was then performed using a panel of DNA probes for chromosomes 3 (MECOM; 3q26), 7 (7q22 & 7q36), 8 (MYC; 8q24), 11 (KMT2A; 11q23), 19 (ERCC1; 19q13 & ZNF443; 19p13) and the X chromosome (DXZ1); all probes were from Kreatech (Leica Biosystems). Sections were analysed and focus stacked images were captured using the Isis imaging system (MetaSystems, Altlussheim, Germany), again, with reference to a stained H&E section. Hybridization with probes for chromosome 3, 7 and 8 confirmed trisomy for each of these chromosomes. Probes for the X chromosome, chromosome 19 and chromosome 11 supported the observation that other chromosomes were disomic (Fig. 3).

## Discussion

This case represents an unusual case of pregnancy failure associated with HM-like villous dysmorphism and a complex triple trisomy and paternal uniparental disomy identified on molecular genotyping studies.

Following early pregnancy failure it is recommended by the Royal College of Obstetricians and Gynaecologists Guidelines [9] that all products of conception are examined histologically since HM cannot be excluded using clinical or sonographic features alone. The presence of villous dysmorphic features including variably sized villi, with patchy hydropic changes, irregular villous outlines and trophoblastic pseudoinclusions in the present case raised the possibility of HM and prompted referral to the regional trophoblastic disease centre. Following histological review villous dysmorphology was confirmed but the morphological findings were not diagnostic for PHM or CHM and hence ancillary studies were undertaken.

Immunostaining of p57^KIP2^, the product of the paternally imprinted gene, *CDKN1C*, shows positive staining of the cytotrophoblast and villous stroma in all pregnancies except for CHM, in which both sets of chromosomes are paternally derived [5], and is thus a useful discriminator in the diagnosis of CHM [3, 5, 10]. However, p57^KIP2^ immunostaining does not discriminate between PHM and non-molar miscarriages. In this setting, molecular genotyping to assist definite diagnosis in cases of suspected gestational trophoblastic disease is now well-reported in clinical practice, either routinely or in selected cases [2–4, 11].

In the present case p57^KIP2^ immunostaining was uniformly negative in the cytotrophoblast and villous stroma suggesting lack of maternal allele expression, as seen most commonly with CHM. Initial genotyping using the AmpFlSTR Identifiler Plus kit showed the tissue to have only a single paternally derived allele for chromosome 11, the chromosome on which the *CDKN1C* gene is located. However, genotyping also demonstrated a normal diploid origin for most other chromosomal pairs with the exception of 3, 7 and 8 for which three alleles were present. FISH was performed to confirm that this was a diploid conception with triple trisomy, rather than a tetraploid with loss of chromosomes 3, 7 and 8. FISH confirmed the presence of 3 copies of chromosomes 3, 7, and 8 and two copies of other chromosomes including chromosome 11. Analysis of additional STR markers on chromosome 11 confirmed that both copies were paternal in origin.

Loss or gain of maternal chromosome 11 has previously been shown to account for unexpected p57^KIP2^ expression in molar tissue. Two cases of CHM with expression of p57^KIP2^ due to retention of a maternally derived chromosome 11 have been described [12, 13], in addition to two PHM in which no expression of p57^KIP2^ was found in either cytotrophoblast or villous stroma due to loss of the maternal copy of chromosome 11 [3, 14]. Two interesting cases of mosaic PHM have also been reported in which loss of chromosome 11, identified by FISH, was seen in the villous stroma of one and the cytotrophoblast of the other with corresponding loss of p57^KIP2^ expression [15]. However, these PHM cases differ from the present study in that, they were triploid conceptions with loss of chromosome 11 while in the present case most chromosome pairs were diploid and biparental.

Abnormal villous morphology, with aberrant p57^KIP2^ and a diploid and biparental genotype is often associated with the rare inherited condition familial recurrent hydatidiform mole [16, 17] in which affected women have a predisposition to CHM. However, in the present case the pathology was not typical of a CHM and three chromosomes were shown to be trisomic rather than disomic.

Trisomy, in an otherwise diploid conception, usually results from meiotic nondisjunction during oogenesis, and is associated with increased maternal age [18]. Double and triple trisomies are rare, triple trisomy accounting for only approximately 0.05% of spontaneous abortions [19]. While triple trisomy involving chromosomes 13, 16, and 21 has been described in tissue from two cases of spontaneous abortion with a differential diagnosis of HM, the parental origin of the chromosomes was not determined [2, 10]. To our knowledge the only other case of triple trisomy of paternal origin previously reported occurred in material from a spontaneous abortion that showed morphological features of a PHM [20]. However, the previous case differed to that in the present study in several respects. While both cases involved trisomy of chromosome 7, the present case involves chromosomes 3, and 8, rather than trisomy of 13 and 20. Secondly, the triple trisomy is likely to have arisen by a different mechanism in each case. In the previous case homozygosity for paternal alleles, suggested an error at paternal meiosis II or mitotic non-disjunction. In the present case genotyping for several STR loci revealed heterozygosity for some, but not all, paternal alleles, making an error at paternal meiosis I a more likely origin for the additional paternal chromosomes. Finally the present case involves paternal disomy for four, rather than three, paternal chromosomes and loss of the maternal chromosome 11 resulting in loss of p57^KIP2^ expression associated with the otherwise similar villous dysmorphic features.

The majority of CHM and PHM are associated with the presence of two copies of the paternal genome. However, duplication of the entire genome is unlikely to be necessary for the development of the morphological features of HM. Studies of cases with unusual genetic constitutions may enable the correlation of different chromosomal abnormalities to specific morphological features of molar pregnancies. It has previously been suggested that trisomy of chromosome 13 is associated with some features of HM seen on ultrasound [21–23]. However, trophoblastic hyperplasia was not found in these cases on morphological examination. Other studies have shown certain chromosomal trisomies, specifically 7 and 15 to show trophoblastic hyperplasia similar to that seen in HM irrespective of parental origin [24]. This is of interest given that trisomy of chromosome 7 was present in both this and the previous case of triple trisomy and that trisomy of chromosome 7 is frequently found in series where genotyping has been performed to aid in the differential diagnosis of products of conception [2, 11] or differential diagnosis of PHM [4, 10]. Other trisomies found in these series include trisomy for 16 and 21, the commonest trisomies in both the two larger series, and trisomy 8,13,18, and X. However, data from these studies is limited to those chromosomes for which markers are included in the kits used for genotyping and some more complex genotypes may be missed. In our study further STR markers were used to investigate the origin of the long and short arm of all chromosomes to demonstrate that chromosomal gain and loss involved the whole chromosome and identify any other chromosomal abnormalities that would not have been identified by the AmpflSTR kit alone. Further studies that include analysis of all chromosomal regions are needed to correlate specific genetic change with recognised pathological abnormalities.

## Conclusions

This case demonstrates several important issues regarding both pathogenesis of disease and clinical practice. First, we confirm that trisomic conceptions may demonstrate villous dysmorphic features, which are similar to CHM and PHM. Secondly, we demonstrate that absent p57^KIP2^ immunostaining indicates loss of maternal gene expression from the p57^KIP2^ region on chromosome 11, rather than CHM. Finally, we confirm the diagnostic importance of selective molecular genetic testing for providing definitive diagnosis in cases of possible HM in which morphological features are non-diagnostic. Whilst routine use of molecular genetic testing is not financially viable for large publically funded services [4], increasing use of molecular genetic testing in clinical practice, in relation to detailed morphological and immunohistochemical findings, will increase our understanding of genotype-phenotype relationships in abnormal pregnancies.

## Consent

Written informed consent was obtained from the patient for publication of this Case Report and any accompanying images. A copy of the written consent is available for review by the Editor-in-Chief of this journal.
